# Assessing the quality of RNA isolated from human breast tissue after ambient room temperature exposure

**DOI:** 10.1371/journal.pone.0262654

**Published:** 2022-01-18

**Authors:** Stella B. Somiari, Susan Shuss, Jianfang Liu, Kimberly Mamula, Amy O’Donnell, Brenda Deyarmin, Jennifer Kane, Amber Greenawalt, Caroline Larson, Sean Rigby, Hai Hu, Craig D. Shriver

**Affiliations:** 1 Chan Soon-Shiong Institute of Molecular Medicine at Windber, Windber, Pennsylvania, United States of America; 2 Murtha Cancer Center Research Program, Uniformed Services University of the Health Sciences, Bethesda, Maryland, United States of America; Nathan S Kline Institute, UNITED STATES

## Abstract

High quality human tissue is essential for molecular research, but pre-analytical conditions encountered during tissue collection could degrade tissue RNA. We evaluated how prolonged exposure of non-diseased breast tissue to ambient room temperature (22±1°C) impacted RNA quality. Breast tissue received between 70 to 190 minutes after excision was immediately flash frozen (FF) or embedded in Optimal Cutting Temperature (OCT) compound upon receipt (T0). Additional breast tissue pieces were further exposed to increments of 60 (T1 = T0+60 mins), 120 (T2 = T0+120 mins) and 180 (T3 = T0+180 mins) minutes of ambient room temperature before processing into FF and OCT. Total exposure, T3 (T0+180 mins) ranged from 250 minutes to 370 minutes. All samples (FF and OCT) were stored at -80°C before RNA isolation. The RNA quality assessment based on RNA Integrity Number (RIN) showed RINs for both FF and OCT samples were within the generally acceptable range (mean 7.88±0.90 to 8.52±0.66). No significant difference was observed when RIN at T0 was compared to RIN at T1, T2 and T3 (FF samples, p = 0.43, 0.56, 0.44; OCT samples, p = 0.25, 0.82, 1.0), or when RIN was compared between T1, T2 and T3. RNA quality assessed by quantitative real-time PCR (qRT-PCR) analysis of *beta-actin* (*ACTB*), *glyceraldehyde-3-phosphate dehydrogenase* (*GAPDH*), *cyclophilin A* (*CYPA*), and *porphobilinogen deaminase* (*PBGD*) transcripts showed threshold values (C_t_) that indicate abundant and intact target nucleic acid in all samples (mean ranging from 14.1 to 25.3). The study shows that higher RIN values were obtained for non-diseased breast tissue up to 190 minutes after resection and prior to stabilization. Further experimental exposure up to 180 minutes had no significant effect on RIN values. This study strengthens the rationale for assessing RIN and specific gene transcript levels as an objective method for determining how suitable RNA will be for a specific research purpose (“fit-for purpose”).

## Introduction

Biobanks contribute to the success of translational research by managing the procurement, processing, annotation, storage, and distribution of biospecimens. These biospecimens, together with their associated clinical data, are critical to our understanding of disease mechanisms and the discovery of new biomarkers. How a sample is handled and processed could affect the quality of RNA extracted from it. RNA degradation could occur due to a number of factors including but not limited to time between surgical resection and stabilization or maintaining tissue samples in suboptimal temperature conditions before stabilization. Such pre-analytical conditions could impact tissue quality and subsequently the RNA derived from these tissue samples.

The time between surgical resection and delivery of a tissue sample to a biobank for stabilization and storage will be collection site dependent. Delivery time could range from less than thirty minutes to a couple of hours depending on the resources available at the collection site to support research. Since biobanks are focused on maximizing the use of available tissue and ensuring that tissue available for research is of the quality needed to perform the analysis they are requested for (“fit-for-purpose”), it becomes important to understand how some pre-analytical conditions, for example, time between receiving and stabilization impact tissue quality.

Using RNA of poor or compromised quality for experiments could generate unreliable or irreproducible data. Currently, RNA quality is commonly assessed by RNA Integrity Number (RIN) using the Agilent 2100 Bioanalyzer technology with a RIN output of 1–10, where 1 represents the most degraded RNA profile and 10 the most intact [1–3].

Although RIN is an objective method for estimating the quality of RNA, it cannot unequivocally predict the integrity of a specific RNA transcript in a sample. Thus, despite the wide use of RIN as a quality control parameter, there is a need to determine the extent to which it can be used as the basis to accept or reject research samples. This is important because not all studies target the same RNA transcripts, and samples with RIN values that do not work for one experiment may be useful for a different experiment. The use of another independent method to accurately establish RNA quality in addition to RIN values may be an option in research and biobanking. A method that can determine the integrity of specific RNA transcripts is quantitative real-time PCR (qRT-PCR). In qRT-PCR, the threshold cycle (C_t_) of each target RNA can be measured, and because the C_t_ value represents the number of cycles required for the fluorescent signal from an amplified template to cross the background threshold, it is inversely proportional to the starting amount of intact target RNA. Greater amounts of starting RNA (or intact/non- degraded RNA) will display earlier threshold crossings, that is, lower C_t_ values. The C_t_ value is a useful determinant of specific RNA integrity and an objective measure of the concentration of intact target RNA. As a “rule of thumb”, C_t_ values of ≤29 indicates the presence of abundant and intact target nucleic acid in the sample, values of 30–37 represent moderate intact target nucleic acid, while values of 38–40 represent minimal intact target nucleic acid [4]. The decision to accept or reject a specimen for research may be best determined by: a) estimating the RIN, and b) determining the concentration of RNA targets that are of specific interest to an experiment.

The objective of the study was to understand how prolonged ambient room temperature exposure after sample receiving affects tissue quality and the derived RNA. To realize this objective, we exposed human breast tissue to increments of one, two and three hours of ambient room temperature. These breast tissue samples had experienced pre-analytical conditions for 70 to 190 minutes post excision, before receipt at the biobank. This situation mimics the everyday scenario of the biobank, thus making this study a true assessment of what happens in real life. The study assessed the quality of RNA derived from these samples based on RIN values and four selected RNA transcripts. The results demonstrate the dual assessment of RIN and threshold cycle values for qualifying research tissue.

## Materials and methods

### Ethical statement

Written informed consent was obtained from the patients who were undergoing reductive mammoplasty surgery. The study protocol (Pro00009470) was approved by Advarra Institutional Review Board.

### Tissue collection and preparation

Breast tissue obtained from right and left breast of three female donors was transported from the surgery suite to the pathology laboratory for clinical diagnosis and from the pathology laboratory to the biobank for stabilization and storage. At the biobank, each breast tissue was trimmed of excess fat and sectioned into multiple pieces. Representative tissue from each donor was then stabilized in two ways to simulate the routine processing and preservation procedures at our biobank: 1) by flash freezing (FF) in liquid nitrogen, and 2) by embedding in OCT prior to storage in -80°C freezers. The time interval from surgical removal of the three mammoplasty cases, to depositing at the biobank is represented by T0, the shortest time to stabilization for each sample. T0 ranged from 70 to 190 minutes and represents the tissue associated condition or pre-analytical/pre-experimental condition. This condition which is outside the biobank’s control could potentially impact tissue quality. Additional processing by FF and OCT embedding for the remaining donor tissue was performed after exposure to room temperature (22°C+/-1°C for an additional 1 hour (T1 samples), 2 hours (T2 samples) and 3 hours (T3 samples). Thus, the total time from surgery to stabilization for the breast tissue samples at T1 to T3 ranged from 250 to 370 minutes. All samples (T0 to T3) were prepared in duplicate and stored at -80°C until RNA isolation. This information is summarized in Table 1.

**Table 1:** Duration of exposure of breast tissue samples before processing and freezing at -80°C.

Exposure Time	Breast Sample 1		Breast Sample 2		Breast Sample 3	
	Right (1-RT)	Left (1-LT)	Right (2-RT)	Left (2-LT)	Right (3-RT)	Left (3-LT)
T0	74 min	89 min	70 min	100 min	185 min	190 min
T1	134 min	149 min	130 min	160 min	245 min	250 min
T2	194min	209 min	190 min	220 min	305 min	310 min
T3	254 min	269 min	250 min	250* min	365 min	370 min

T0 = Initial exposure from excision to processing and freezing (pre-analytical/pre-experimental factor); T1 = T0+60 mins; T2 = T0+120 mins; T3 = T0+180 mins. *T3 = T0+150 minutes. Ambient Room Temperature was 22±1°C.

### RNA isolation from FF and OCT-embedded tissue

Total RNA was isolated from 90-120mg of each FF sample using the RNeasy Lipid Mini Kit (QIAGEN, Valencia, CA) following the manufacturer’s protocol. For each OCT sample, RNA was isolated from 5–10 sections (20μm thickness) with a total weight of approximately 400mg. The OCT sections were transferred into five microcentrifuge tubes and RNA isolation carried out with the RNeasy Lipid Tissue Mini Kit (QIAGEN, Valencia, CA) following the manufacturer’s instructions. RNA concentration (ng/μL) and purity (A_260/280_) were determined for each RNA sample using the Nanodrop 1000 (ThermoFisher Scientific, Wilmington, DE). All RNA samples were stored at -80°C until RIN assessment and qRT-PCR.

### Determination of RNA quality/integrity

The quality or integrity of the RNA isolated from the samples was determined by measuring the RIN and quantifying the level of four RNA transcripts by qRT-PCR. The RIN was determined with the 2100 Bioanalyzer using the RNA 6000 Nano Kit (Agilent Technologies, Santa Clara, CA). Quantitative Real-Time PCR was performed with the iCycler iQ Real-Time PCR Detection System using the iQ SYBR Green Supermix Kit (Bio-Rad, Hercules, CA). The qRT-PCR targeted the RNA transcripts for the following: *beta-actin (ACTB)*, *glyceraldehyde-3-phoshate dehydrogenase (GAPDH)*, *cyclophilin A (CYPA)* and *porphobilinogen deaminase (PBGD)* using the forward and reverse primers presented in Table 2. The chosen targets are housekeeping or reference genes expressed constitutively by different cell types and their expression level is expected to be similar in all samples (stable), that is, not showing changes under experimental conditions or disease state [5].

**Table 2:** Primer sequences for qRT-PCR targeting *ACTB*, *GAPDH*, *CYPA* and *PBGD*.

HKG Name	Forward Primer Sequence	Reverse Primer Sequence	Fragment Size (bp)
*ACTB*	CTCTTCCAGCCTTCCTTCCT	AGCACTGTGTTGGCGTACAG	116
*GAPDH*	CTCTGCTCCTCCTGTTCGAC	ACGACCAAATCCGTTGACTC	112
*CYPA*	GGATGGCAAGCATGTGGTG	TGTCCACAGTCAGCAATGG	123
*PBGD*	AGGATGGGCAACTGTACCTG	ACCAACTGTGGGTCATCCTC	133

To synthesize the cDNA, 1μg of total RNA was reverse transcribed using the High Capacity cDNA Reverse Transcriptase (RT) Kit per the manufacturer’s protocol (ThermoFisher Scientific, Foster City, CA) using a final reaction volume of 20μL. The final qRT-PCR reaction volume was 50μL, consisting of 2μL of cDNA sample, 1μL of each primer mix and 25μL of iQ SYBR Green Supermix and 22μL nuclease free water. A 40-cycle protocol was run for all of the reactions as follows: denaturation for 1 minute at 95°C, primer annealing for 1 minute at 55–68°C and extension for 1 minute at 72°C. All samples were run in duplicate, and No Amplification (NAC) and No Template (NTC) Controls were included.

### Data analysis

Yield, purity, and RIN of RNA from the extended exposure time points were compared to the baseline of each breast sample. Also compared were C_t_ values at the different time points to determine how extended exposure to room temperature affected the level of the RNA transcripts of *ACTB*, *GAPDH*, *CYPA* and *PBGD* and the impact of RIN on C_t_ values. Based on the experimental design, data were compared using either paired t-test or paired Wilcoxon signed rank test depending on the normality of the data. Data were further analyzed using a mixed model of random effects and fixed effects. If the assumption of normality for the mixed model was not met, and the log transformation of the data was normal, then log-normal data were used for the mixed model analysis. All results were considered statistically significant when p<0.05 using a two-tailed test.

## Results

### Total RNA quality metrics for Flash Frozen (FF) and OCT-embedded breast tissue

**Fig 1:**
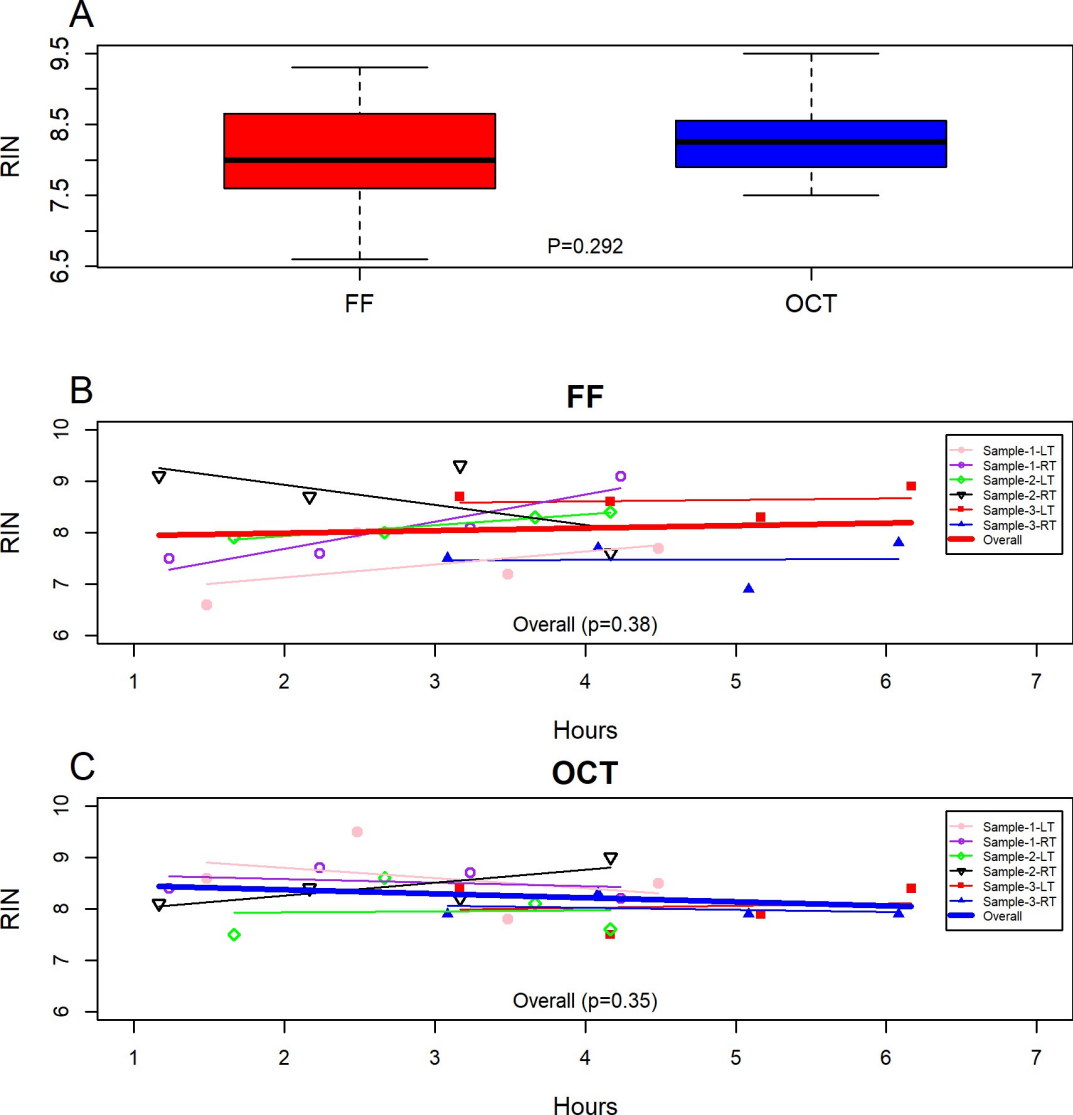
RNA quality based on RIN for FF and OCT embedded samples. **A**- Box plot of average RIN for FF and OCT. Change in average RIN in relation to extended room temperature exposure for **B**- FF samples and **C-** OCT samples.

Mean RIN values (± standard deviation) for the samples were between 7.88±0.90 and 8.25±0.65 for the FF and between 8.10±0.33 and 8.52±0.66 for the OCT samples (Table 3). OCT samples had slightly higher RIN values compared to the FF samples but no significant difference was observed (p = 0.292, Fig 1A). For RNA purity (A_260/280_ absorbance ratio), mean values were between 2.08 and 2.12 for the FF samples and between 2.0 and 2.08 for the OCT samples indicating high purity or limited contamination with genomic DNA or proteases [6–9]. RNA recovery was good for all samples, with mean RNA yield between 6.63μg and 14.15μg for the FF samples and between 2.42μg and 3.41μg for the OCT samples.

**Table 3:** Means and medians of the RIN, purity, and yield from RNA isolated from breast tissue processed as flash frozen (FF) or embedded in optimal cutting temperature (OCT) medium.

	RNA metrics	T0*		T1		T2		T3**	
		Mean±SD	Median	Mean±SD	Median	Mean±SD	Median	Mean±SD	Median
FF N = 6	RIN	7.88±0.90	7.70	8.10±0.46	8.00	8.02±0.86	8.20	8.25±0.65	8.10
	A260/280	2.08±0.05	2.08	2.12±0.02	2.12	2.09±0.04	2.09	2.10±0.06	2.12
	Yield (μg)	6.63±4.22	5.19	14.15±16.82	6.80	9.02±9.60	6.28	12.84±8.61	14.20
OCT N = 6	RIN	8.15±0.40	8.25	8.52±0.66	8.50	8.10±0.33	8.00	8.27±0.49	8.30
	A260/280	2.08±0.20	2.07	2.08±0.09	2.08	2.08±0.11	2.10	2.00±0.11	2.05
	Yield (μg)	2.92±2.90	1.70	2.42±1.79	1.64	2.74±2.07	1.97	3.41±2.10	2.80

*T0 = pre-analytical/pre-experimental factor (time between surgical resection to stabilization at the biobank; ranging from 70 to 190 minutes for the 6 samples).

T1 = (T0+60 minutes).

T2 = (T0+120 minutes).

T3 = (T0+180 minutes).

** For 1 case (FF and OCT), T3 = T0+150 minutes.

### Effect of extended ambient room temperature on the RNA quality

RNA quality at T0, which is the status of the tissue at receipt, was not significantly different from the RNA obtained after an additional 60 minutes of exposure at ambient temperature (T0 vs T1) for FF (p = 0.43) and OCT (p = 0.25; Table 4). Additional ambient temperature exposure of the tissue up to 180 minutes (T2 and T3) also had no significant difference on RNA quality based on RIN for either FF or OCT processed tissue (Table 4).

**Table 4:** Paired test of comparison of RIN at T0 and after additional ambient temperature exposure (T1, T2, and T3) before flash freezing and OCT embedding.

	p-values					
	T0 vs T1	T0 vsT2	T0 vsT3	T1 vsT2	T1 vs T3	T2 vsT3
Change in RIN-FF (N = 6)	0.430	0.555	0.435	0.762	0.686	0.438*
Change in RIN-OCT (N = 6)	0.25	0.82	1.00*	0.21	0.49	0.52

*Paired Wilcoxon signed rank test was used due to the data not passing normality test; all others are paired t-test.

The data were then analyzed together for FF and OCT samples respectively using mixed effects modeling with time as a fixed effect and each sample as a random effect to further check if tissue exposure time has an effect on the RNA quality. Based on the analysis, RIN for FF and OCT samples were not significantly affected by exposure time (p = 0.38 and 0.35; Fig 1B and 1C). These data indicate that RNA was not degraded after an additional 180 minutes at ambient temperature.

### Effect of ambient room temperature on the RNA transcripts encoding *ACTB*, *GAPDH*, *CYPA* and *PBGD*

**Fig 2:**
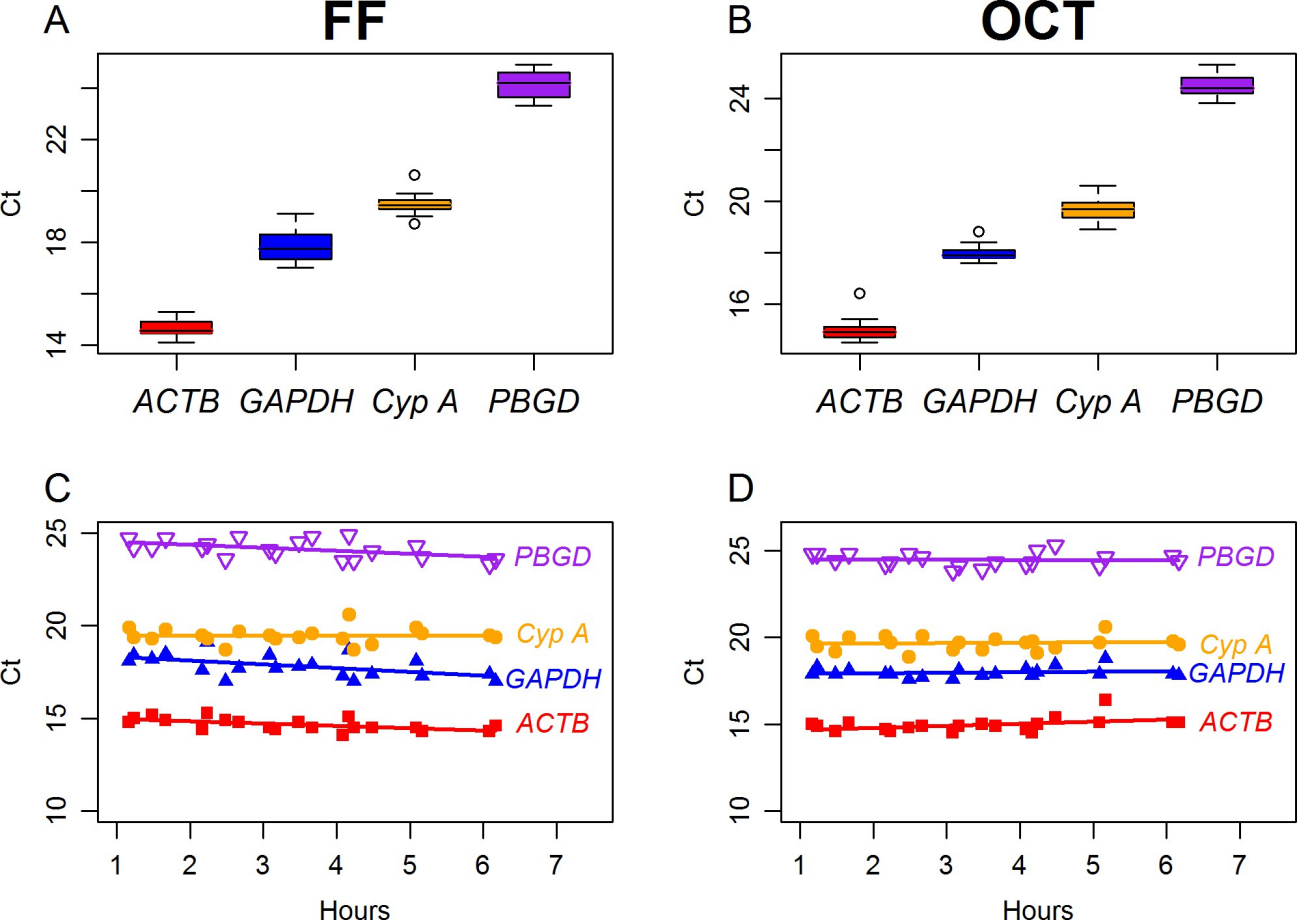
Average Ct values of 4 gene transcript for FF and OCT embedded samples. Boxplot of average C_t_ values for *ACTB*, *GAPDH*, *CYPA*, and *PBGD* in **A**- FF samples and **B**- OCT samples. Change in average C_t_ value for *ACTB*, *GAPDH*, *CYPA*, and *PBGD* in relation to extended room temperature exposure for **C**- FF samples and **D**- OCT samples.

The effect of extended exposure at room temperature on transcripts of *ACTB*, *GAPDH*, *CYPA* and *PBGD* was determined by qRT-PCR. The mean C_t_ values for FF samples ranged from 14.1 to 15.3 for *ACTB*, 17 to 19.1 for *GAPDH*, 18.7 to 20.6 for *CYPA* and 23.3 to 24.9 for *PBGD*. The mean C_t_ values for OCT samples ranged from 14.5 to 16.4 for *ACTB*, 17.6 to 18.8 for *GAPDH*, 18.9 to 20.6 for *CYPA* and 23.8 to 25.3 for *PBGD*. Threshold values for *PBGD* in both FF and OCT samples were observed to be consistently higher compared to *ACTB*, *GAPDH*, and *CYPA* (Fig 2A–2D) indicating their stability compared to *PBGD* in the breast tissue under the different conditions.

Mixed model of random and fixed effects was performed to determine the effect of extended exposure to room temperature and RIN on C_t_ values. For this analysis, each sample was treated as a random effect; hours at room temperature and RIN were treated as fixed effects. We observed that for the FF samples, C_t_ was significantly reduced with increased time of exposure to room temperature for *GAPDH* (p = 0.036; Table 5) with a trend toward significance for *ACTB* (p = 0.065). This observation is opposite to the expectation since RNA degradation would be expected from prolonged room temperature exposure, and this would be associated with increase in Ct values. However, C_t_ values for OCT samples were not significantly affected by the length of time the samples were exposed at room temperature [*CYPA* (p = 0.177), *GAPDH* (p = 0.407), *PBGD* (p = 0.521) and *ACTB* (p = 0.102); Table 5] which could imply that prolonged exposure had no impact on RNA quality.

**Table 5:** Summarized results for mixed model analyses of C_t_ from FF, OCT, and FF + OCT; p-values at 0.05 level of significance.

	C_t_	Normality Test p-value	RIN		Time (minutes)		Format (OCT vs FF)	
			Estimate	P-value	Estimate	P-value	Estimate	P-value
FF (n = 20)	*ACTB*	0.4792	-0.223	0.022*	-0.0013	0.065		
	*GAPDH*	0.5394	-0.628	0.0009*	-0.0026	0.036*		
	*CYPA*	0.1343	-0.482	2.2e-05*	0.0009	0.119		
	*PBGD*	0.306	-0.371	0.009*	-0.0009	0.307		
OCT (n = 20)	*ACTB* ^*$*^	0.0003*	-0.333	0.084	0.0017	0.094		
	*ACTB* ^*#*^	0.372	-0.194	0.102	0.0009	0.123		
	*GAPDH* ^*$*^	0.017*	-0.164	0.273	0.0003	0.645		
	*GAPDH* ^*#*^	0.198	-0.097	0.407	-0.00016	0.792		
	*CYPA*	0.923	-0.232	0.177	9.5e-05	0.915		
	*PBGD*	0.805	0.132	0.521	-6.2e-05	0.952		
FF + OCT (n = 40)	*ACTB* ^*$*^	0.0008*	-0.298	0.005*	0.00005	0.944	0.394	0.001*
	*ACTB* ^*#*^	0.458	-0.217	0.007*	-0.0002	0.709	0.295	0.002*
	*GAPDH*	0.454	-0.352	0.004*	-0.0014	0.067	0.270	0.056
	*CYPA*	0.369	-0.316	0.0002*	0.0004	0.517	0.317	0.0004*
	*PBGD*	0.454	-0.098	0.419	-0.0008	0.340	0.360	0.013*

^$^Data with one outlier for the data values, the log transformation of the data was normal, and log-normal data were used for mixed model analysis.

^#^Data when the single outlier is removed.

*Shows significant value.

For the association of C_t_ with RIN, for the FF samples, we observed that when RIN increased, C_t_ values significantly decreased for *CYPA* (p<0.0001), *GAPDH* (p = 0.0009), *PBGD* (p = 0.009) and *ACTB* (p = 0.022) (Table 5). Although OCT samples showed no significance for this trend [*CYPA* (p = 0.177), *GAPDH* (p = 0.407), *PBGD* (p = 0.521) and *ACTB* (p = 0.102); Table 5)] when data for FF and OCT are combined, C_t_ values significantly decrease with increasing RIN for *ACTB* (p = 0.007), *GAPDH* (p = 0.004), and *CYPA* (p = 0.0002) except for *PBGD* (p = 0.419; Table 5). This finding meets the expectation of a negative association of C_t_ and RIN. The combined data for FF and OCT shows that compared to FF samples, OCT samples had significantly higher C_t_ values for *ACTB* (p = 0.002), *CYPA* (p = 0.0004), *PBGD* (p = 0.013), with a trend toward significance for *GAPDH* (p = 0.056) (Table 5).

## Discussion

In this study, we determined how RNA quality from non-diseased breast tissue (reductive mammoplasty) is impacted by prolonged exposure to ambient room temperature (22°C±1°C) before flash freezing or embedding in OCT. It is important to note that T0 (pre-analytical/pre-experimental time) represents the time between tissue excision to stabilization at the biobank and the shortest T0 was 70 minutes while the longest T0 was 190 minutes (Table 1). The biobank had no control over T0 and the condition that the tissue was exposed to prior to receipt. At the biobank, additional pieces of the breast tissue samples were further exposed to ambient temperature for 1, 2 and 3 hours before flash freezing or embedding in OCT. This represented the experimental manipulation of the tissue with the longest T3 exposure time of 370 (that is, T0 of 190 minutes plus the additional 180 minutes at room temperature before stabilization; Table 1). Generally, the acceptable cutoff range of RIN recommended for successful gene expression studies is RIN ≥6 [1, 10]. RIN values for all samples at all time points for FF and OCT ranged between 6.6 and 9.5 indicating relatively intact RNA.

No significant difference was observed when RIN at T0 (time from resection to delivery at the biobank) was compared to T1, T2 and T3 (Table 4) indicating that the RNA quality at T0 was similar to that at T1, T2 and T3 (Table 3) for all breast samples. Thus, the additional exposure of the breast tissue to ambient temperature for up to 180 minutes (3 hours) after receiving the sample at the biobank seemed to have no adverse impact on the quality of the breast tissue and the RNA extracted from it. Our findings are in line with earlier studies that compared RNA stability in different organs at different temperature conditions and ischemia time [11–13]. Liu et al. (2002) and Jewell et al. (2013) reported that RNA is stable in lung and kidney tissue up to 4 and 5 hours respectively after surgical resection [11, 12]. Studies have also shown that cold ischemia times of 1–6 hours do not affect RNA quality based on RIN assessment [12, 14–16].

Although the RIN values obtained in our study were indicative of relatively good quality RNA, it was important to determine if specific RNA transcripts were intact in the FF and OCT samples. Using qRT-PCR, we investigated transcripts of *ACTB*, *GAPDH*, *CYPA* and *PBGD*. We observed the lowest C_t_ value of 14.1 for *ACTB* (FF) and the highest C_t_ value of 24.9 for *PBGD* (OCT). The C_t_ value represents the number of replication cycles required to produce fluorescent signal above the threshold, and the lower the C_t_ values, the more abundant the transcript at the start of the analysis. Threshold values of ≤29 indicate the presence of abundant intact target nucleic acid in a sample [4]. Based on the observed C_t_ values, we conclude that the gene expression levels of the housekeeping genes were high when samples were received at the biobank and remained high during the additional experimental exposure to room temperature before stabilization by FF and OCT embedding.

As expected, we observed increased RIN with decreased C_t_ value for *ACTB*, *GAPDH*, *CYPA*, and *PBGD* in the FF samples; for the OCT samples, this relationship was not significant, but the same trend was observed except for *PBGD* (Table 5). Combining data for FF and OCT, we observed a trend of increasing RIN and decreasing C_t_ values which was significant for *ACTB*, *GAPDH*, and *CYPA* but not for *PBGD* (Table 5). This result for *PBGD* was unexpected, but this gene did show consistently higher C_t_ values for both FF (23.3 to 24.9) and OCT (23.8 to 25.3) samples compared to those for *ACTB*, *GAPDH*, and *CYPA* which were below 21 (Fig 2). There was a consistent trend of lower C_t_ values for *ACTB*, followed by *GAPDH*, and *CYPA* for both OCT and FF samples (Fig 2). Although the consistently higher C_t_ values for *PBGD*, was maintained in both FF and OCT through all the experimental conditions as expected for a reference gene, the consistent lack of significance for OCT and the combined OCT and FF data may reflect other underlying factors that may make *PBGD* unsuitable as a reference gene for breast tissue. The importance of experimental validation of appropriate housekeeping genes to determine the most suitable genes for any given experiment has been previously reported [5].

Overall, RINs did not change with time, but there was noticeable RIN increase with time for breast samples 1-RT in FF and 2-RT in OCT. Such nominal observation, if proven true, contradicts the expectation that in general RNA should degrade with time. Similarly in RT-PCR analysis, C_t_ of GAPDH significantly decreased with time (Table 5). Interestingly, increased/upregulated gene expression levels over time have been observed in colorectal and ovarian cancer [14, 17]. These unexpected results may reflect on the complex biological processes taking place after a tissue is removed from the human body before stabilization and storage which are the uncontrolled pre-analytical variables [18–21].

We would caution that this study focused on extended room temperature exposure after samples were received. Therefore, our results should not be misinterpreted as evidence that RNA quality does not change from the time of sample resection up until several hours before stabilization. We did not study the possible changes at resection (0 minutes) to time at receipt (T0) and studies have reported changes that have occurred less than 20 minutes after resection (20). Our study also focused on normal breast tissues, and our observations may or may not be extrapolated to diseased breast tissue. Studies have shown tumor samples, for example, from colorectal cancer to exhibit higher but less variable RIN values compared to normal samples less than 20 minutes after resection [17]. Also, our RT-PCR study focused on housekeeping genes, but other studies have reported the impact of pre-analytical conditions of tissue in relation to the expression of cancer-related and cell regulatory genes [22, 23]. Finally, other factors that are unassociated with pre-analytical variables can impact RNA integrity such as RNA processing protocols, the specific kits used for RNA isolation versus organ types (for example fatty breast tissue) and how well personnel performing the isolations adhere to procedures [24]. All of these aspects will need to be considered independently to provide insights to researchers regarding optimal collection requirements that meet their specific research needs.

In conclusion, using normal breast tissue received in our biobank from a local medical center with a receiving time of 70–190 minutes after sample resection, which reflects a real-life sample receiving experience, we show that the RNA quality determined by RIN and RT-PCR of four housekeeping genes was high at receipt and after 1–3 hours of additional room temperature exposure (mean RINs, between 7.88±0.90 and 8.52±0.66; mean C_t_ ranges 14.1 to 25.3). The results presented here may guide the biobank in its sample processing workflow design including opportunity to triage samples for processing as they are received based on knowledge of the time between resection and receipt at the biobank.

## Supporting information

S1 Table Excel sheet of sample RNA parameters and C_t_ values. Details of all RNA parameters for both Flash Frozen (FF) and Optimal Cutting Temperature (OCT) processed samples at different time points which include—RIN values, concentration (ng/ul), yield (ug) and 260/280 ratios and sample C_t_ values for *ACTB*, *GAPDH*, *CYPA* and *PBGD*.
(XLSX)
